# The A-VIVO score: a novel prognostic tool for predicting mortality in patients with sepsis

**DOI:** 10.1007/s11845-026-04475-z

**Published:** 2026-06-10

**Authors:** Öztürk Taşkın, Ufuk Demi̇r, Veysel Garani Soylu, Ayşe Yılmaz, Büşra Tanyıldızı Küçük

**Affiliations:** 1 Department of Anesthesiology and Reanimation, Faculty of Medicine, Kastamonu University, Kastamonu, Türkiye; 2 Intensive Care Clinic, Faculty of Medicine, Kastamonu University, Kastamonu, Türkiye; 3 Department of Anesthesiology and Reanimation, Faculty of Medicine, İzmir Tınaztepe University, İzmir, Türkiye

**Keywords:** Sepsis, Mortality, Intensive care, prognostic score, A-VIVO, Infection

## Abstract

**Background:**

Sepsis is a life-threatening clinical syndrome associated with high morbidity and mortality in patients admitted to intensive care units. Early prediction of mortality risk is essential for timely interventions and optimal use of critical care resources. Although various prognostic scoring systems are available, many are complex or not easily applicable in daily clinical practice. Therefore, simple and reliable tools for early risk stratification are needed.

**Aims:**

The aim of this study was to evaluate the prognostic performance of the A-VIVO score in predicting early mortality in patients with sepsis.

**Methods:**

This retrospective, single-center observational study reviewed electronic medical records of adult patients diagnosed with sepsis according to the Sepsis-3 criteria and admitted to the intensive care unit between January 2020 and January 2025. Patients aged 18 years or older who were monitored for at least 48 h were included. Patients with missing data or unavailable mortality outcomes were excluded. Patients were classified as survivors or non-survivors based on 30-day mortality. The A-VIVO score was calculated using eight clinical and laboratory parameters, each scored from 0 to 2 points.

**Results:**

A total of 683 patients were included. Of these, 400 patients (58.6%) died within 30 days, while 283 patients (41.4%) survived. The A-VIVO score was significantly higher in non-survivors and showed a strong association with mortality.

**Conclusions:**

The A-VIVO score is a simple and practical tool for predicting mortality in patients with sepsis and may support early clinical decision-making.

**Supplementary Information:**

The online version contains supplementary material available at https://doi.org/10.1007/s11845-026-04475-z.

## Introduction

Sepsis is a life-threatening organ dysfunction resulting from a dysregulated host response to infection and is one of the leading causes of morbidity and mortality in intensive care units (ICUs). The current definition of Sepsis-3 criteria emphasizes the importance of organ dysfunction in early diagnosis of sepsis and prediction of prognosis [1]. Worldwide, sepsis mortality rates are reported to be between 25 and 40%, and this rate increases further in the presence of septic shock [1, 2].

Predicting mortality risk in intensive care patients is critical for clinical decision-making processes, guiding treatment strategies, and optimizing resource utilization. For this purpose, various clinical scoring systems such as Acute Physiology and Chronic Health Evaluation II (APACHE II), Sequential Organ Failure Assessment (SOFA), and Simplified Acute Physiology Score II (SAPS II) have been developed and are widely used. While these scores are powerful tools for predicting mortality, they have significant limitations, such as relying on numerous clinical and laboratory parameters, requiring complete data from the first 24 h, being time-consuming to calculate, and not routinely monitoring all necessary parameters in some centers [3, 4]. The practical applicability of these scores may be limited, especially in intensive care units with high patient loads and limited resources.

For these reasons, there is a growing need for new scoring systems based on simpler, faster, objective, and readily accessible parameters for predicting mortality in sepsis patients [5]. Recent studies have shown that age, mean arterial pressure (MAP), level of consciousness (Glasgow Coma Score, GCS), inflammatory markers (especially neutrophil-lymphocyte ratio [NLR]), need for invasive or non-invasive mechanical ventilation, and creatinine, bilirubin, and lactate levels reflecting renal and hepatic function are significantly associated with sepsis mortality [6–8].

The impact of MAP targets on prognosis in septic shock is particularly emphasized due to their critical role in maintaining organ perfusion [6]. Similarly, simple and inexpensive inflammation markers such as NLR have been shown to be independent predictors of mortality in patients with sepsis [7]. All of these parameters consist of clinical and laboratory data routinely obtained in intensive care units and can be used without requiring additional testing, time, or cost.

The A-VIVO score (Age, Vital signs [MAP and GCS], Inflammation, Ventilation, Organ function), was developed as a simplified clinical tool based on routinely available parameters for 30-day mortality risk assessment in sepsis. The aim of this study was to evaluate its prognostic performance in predicting 30-day mortality in intensive care unit patients.

## Methods

This study was designed as a retrospective, single-center, observational study to evaluate the prognostic performance of the A-VIVO score for 30-day mortality prediction in patients with sepsis admitted to the intensive care unit.

**Fig. 1:**
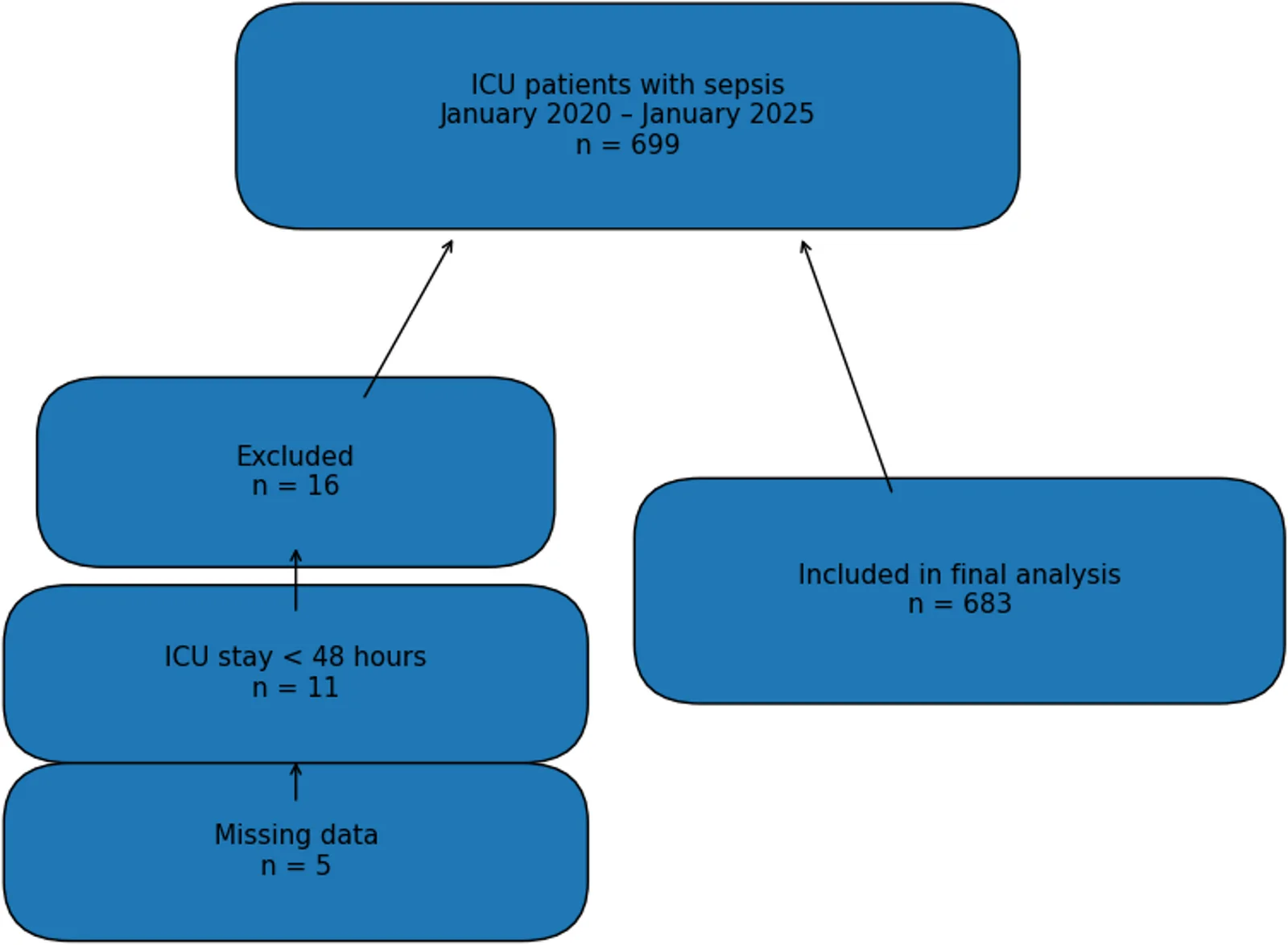
Flowchart of patient selection for the study

Electronic medical records of patients diagnosed with sepsis according to the Sepsis-3 criteria and monitored in the intensive care unit between January 2020 and January 2025 were retrospectively reviewed. Patients aged ≥ 18 years who were monitored for at least 48 h were included. If a patient had multiple intensive care unit admissions, only the first admission was considered. Patients with missing mortality data (*n* = 11) or insufficient data for score calculation (*n* = 5) were excluded. A total of 683 patients were included in the study (Fig. 1). The study protocol was approved by the Kastamonu University Non-Interventional Clinical Research Ethics Committee (Approval No: 2025-78) and conducted in accordance with the Declaration of Helsinki.

### Data collection

Patients were categorized into survivors and non-survivors based on 30-day mortality. Demographic characteristics, comorbidities, clinical findings, laboratory parameters, and disease severity scores were obtained from electronic medical records. The patients’ age, gender, comorbidities (diabetes mellitus, hypertension, cerebrovascular disease, chronic respiratory disease, cardiovascular disease, and chronic kidney disease), clinical parameters (MAP, GCS), laboratory parameters (creatinine, albumin, total bilirubin, C-reactive protein (CRP), CRP/albumin ratio (CAR), lactate), complete blood count parameters (leukocytes, hemoglobin, hematocrit, platelets, Red Cell Distribution (RDW) neutrophil, lymphocyte, monocyte counts, NLR), and disease severity scores (A-VIVO, APACHE II, SOFA and SAPS II and NEWS) were recorded.

### Development of the A-VIVO score

#### Rationale for variable selection

Each variable included in the A-VIVO score was selected based on its established association with sepsis severity and mortality and its representation of key pathophysiological domains.

Age has been consistently identified as an independent predictor of mortality in critically ill patients. Mean arterial pressure (MAP) reflects hemodynamic stability and tissue perfusion and plays a central role in the definition and management of septic shock [1, 2]. Glasgow Coma Scale (GCS) represents neurological status and is commonly used to assess disease severity and organ dysfunction in critically ill patients [9].

The neutrophil-to-lymphocyte ratio (NLR) was included as a marker of systemic inflammation and immune response and has been shown to be associated with prognosis in sepsis [7, 10]. Lactate was selected as a surrogate marker of tissue hypoperfusion and metabolic stress and is a well-established predictor of mortality in sepsis [2, 11].

Creatinine and total bilirubin were included to reflect renal and hepatic dysfunction, respectively, both of which are key components of organ failure assessment systems such as the SOFA score [9]. The need for respiratory support was incorporated as an indicator of respiratory failure and overall disease severity.

### Determination of cut-off values

Cut-off values for each parameter were defined using a combination of:

- clinically accepted thresholds reported in previous studies, and.
- receiver operating characteristic (ROC) curve analysis within the study cohort to optimize mortality discrimination.

The A-VIVO score was developed using variables that are strongly associated with sepsis mortality, represent key pathophysiological domains, and are routinely available in clinical practice.

The score consists of eight parameters (Table 1):

**Table 1:** Components and Scoring Criteria of the A-VIVO Score

Parameter	Dataset variable	0 Points	1 Point	2 Points
Age	V-AGE	< 65 years	65–79 years	≥ 80 years
Mean Arterial Pressure (MAP)	V-MAP	> 65 mmHg, no vasopressor	Vasopressor required, MAP > 65 mmHg	Refractory hypotension, MAP < 65 mmHg
Level of Consciousness (GCS)	V-GCS	≥ 13	9–12	≤ 8
Respiratory Support	V-RESPIRATORY SUPPORT	No oxygen or low-flow O₂ (O_2_≤ 5/Lt)	High-flow O₂ or non-invasive ventilation (O_2_≥ 6/Lt -NIV)	Invasive mechanical ventilation
Creatinine (mg/dL)	V-CREATININE	< 1.2	1.2–2.0	> 2.0
Total Bilirubin (mg/dL)	V-TOTAL BILIRUBIN	< 1.2	1.2–2.5	> 2.5
Neutrophil-to-Lymphocyte Ratio (NLR)	V-NLR	< 5	5–10	> 10
Lactate (mmol/L)	V-LACTATE	< 2	2–4	> 4

- Age.
- Mean arterial pressure (MAP).
- Glasgow Coma Scale (GCS).
- Need for respiratory support.
- Creatinine.
- Total bilirubin.
- Neutrophil-to-lymphocyte ratio (NLR).
- Lactate.

### Calculation of the A-VIVO score

#### All variables used to calculate the A-VIVO score were obtained at the time of intensive care unit admission, based on routinely collected clinical and laboratory data

The scoring system, including variable categories and corresponding point allocations, is presented in Table 1.

Each parameter was assigned a score ranging from 0 to 2 points, reflecting increasing severity.

Equal weighting of variables was intentionally used to:

- simplify calculation,
- enhance bedside applicability,
- and ensure reproducibility across different clinical settings.

The total A-VIVO score ranges from 0 to 16, calculated as:

#### A-VIVO score = Age + MAP + GCS + respiratory support + creatinine + total bilirubin + NLR + lactate

Higher scores indicate greater mortality risk.

### Statistical analysis

Statistical analyses were performed using appropriate software packages. The distribution of continuous variables was evaluated using visual methods and appropriate normality tests. Continuous variables were presented as median (interquartile range), and categorical variables as number and percentage. In comparisons between surviving and non-surviving patients, the Mann–Whitney U test was used for continuous variables, the Chi-square test for categorical variables, or Fisher’s exact test where appropriate.

The mortality-separating performance of A-VIVO, APACHE II, SOFA, and SAPS II scores was evaluated using receiver operating characteristics (ROC) curve analysis. Area Under the Curve (AUC) and 95% confidence intervals were calculated for each score. AUC values were compared using the DeLong test. The optimal cutoff value for the A-VIVO score was determined using the Youden index. Selectable, specific, positive, and negative predictive values, along with probability amounts, were calculated for this cutoff value. The association between the A-VIVO score and mortality was assessed using univariable logistic regression analysis, and the results were reported as odds ratios (OR) with 95% confidence intervals. An APACHE II-like risk classification was created based on the A-VIVO score, and observed and model-derived predicted mortality rates were calculated according to score ranges. The relationship between A-VIVO and APACHE II scores was evaluated using Spearman correlation analysis. A statistical significance level of *p* < 0.05 was accepted.

## Results

A total of 683 patients diagnosed with sepsis and monitored in the intensive care unit were included in the study. 400 patients (58.6%) died during the study period, while 283 (41.4%) survived.

Demographic, clinical, laboratory, and disease severity characteristics of patients are presented in Table 2 according to survival status. No significant age difference was found between surviving and deceased patients (median 77 [69–83.5] and 76 [66–84] years, respectively; *p* = 0.673). Male sex was significantly higher in the surviving group (*p* < 0.001).

**Table 2:** Demographic, clinical, laboratory, and severity characteristics of the study population

Parameter	All patients (n=683)	Survivors (n=283)	Non-survivors (n=400)	p value
Sex (male), n (%)	304 (44.5)	243 (85.9)	61 (15.3)	<0.001
Age, years	77.00 (68.00–84.00)	77.00 (69.00–83.50)	76.00 (66.00–84.00)	0.673
Any comorbidity, n (%)	493 (72.2)	191 (67.5)	302 (75.5)	0.019
Diabetes mellitus, n (%)	159 (23.3)	52 (18.4)	107 (26.8)	0.014
Hypertension, n (%)	246 (36.0)	85 (30.0)	161 (40.2)	0.008
Cerebrovascular disease, n (%)	109 (16.0)	34 (12.0)	75 (18.8)	0.018
Chronic respiratory disease, n (%)	195 (28.6)	42 (14.8)	153 (38.2)	<0.001
Cardiovascular disease, n (%)	269 (39.4)	63 (22.3)	206 (51.5)	<0.001
Chronic kidney disease, n (%)	131 (19.2)	34 (12.0)	97 (24.2)	<0.001
Mean arterial pressure, mmHg	63.00 (58.00–71.00)	67.00 (60.00–72.00)	60.00 (55.00–70.00)	<0.001
Glasgow Coma Scale	10.00 (7.00–13.00)	13.00 (10.00–15.00)	8.00 (5.00–10.00)	<0.001
Creatinine, mg/dL	1.45 (0.85–2.35)	1.10 (0.75–1.80)	1.77 (0.98–2.90)	<0.001
Albumin, g/dL	2.80 (2.40–3.20)	3.00 (2.60–3.40)	2.60 (2.30–3.00)	<0.001
Total bilirubin, mg/dL	0.95 (0.60–1.60)	0.80 (0.60–1.00)	1.11 (0.66–2.30)	<0.001
C-reactive protein, mg/L	148.0 (78.0–242.0)	124.0 (63.0–198.0)	176.0 (97.0–284.0)	<0.001
CAR	52.7 (24.8–92.4)	39.8 (18.6–69.4)	69.2 (36.5–115.8)	<0.001
White blood cell count	13.2 (9.2–18.6)	11.4 (8.6–15.9)	14.9 (10.7–20.6)	<0.001
Hemoglobin, g/dL	10.5 (9.1–11.9)	11.2 (10.0–12.4)	9.9 (8.7–11.1)	<0.001
Hematocrit, %	32.0 (28.0–36.0)	34.0 (31.0–37.0)	30.0 (27.0–34.0)	<0.001
Platelet count	185 (118–262)	212 (154–285)	162 (98–238)	<0.001
RDW	15.7 (14.2–17.5)	14.9 (13.8–16.3)	16.6 (15.0–18.4)	<0.001
Neutrophil count	10.9 (7.4–15.8)	9.0 (6.4–12.7)	12.6 (8.9–17.9)	<0.001
Lymphocyte count	0.9 (0.6–1.4)	1.1 (0.8–1.6)	0.7 (0.5–1.1)	<0.001
Monocyte count	0.7 (0.4–1.0)	0.8 (0.5–1.1)	0.6 (0.4–0.9)	<0.001
Neutrophil-to-lymphocyte ratio	9.10 (5.80–15.60)	6.70 (4.30–9.90)	12.60 (7.90–20.10)	<0.001
Lactate, mmol/L	2.70 (1.80–4.00)	2.50 (1.90–3.10)	2.90 (1.70–5.10)	<0.001
A-VIVO score	8.00 (6.00–10.00)	6.00 (5.00–8.00)	10.00 (7.00–11.00)	<0.001
APACHE II	26.00 (20.00–33.00)	20.00 (17.00–25.00)	31.00 (25.00–38.00)	<0.001
SAPS II	46.00 (35.00–61.00)	36.00 (30.00–46.00)	52.00 (42.00–72.00)	<0.001
SOFA	7.00 (4.00–10.00)	5.00 (3.00–7.00)	9.00 (7.00–12.00)	<0.001
NEWS	8.00 (6.00–10.00)	7.00 (5.00–9.00)	9.00 (7.00–11.00)	<0.001

A-VIVO Score; Age, Vital signs (MAP and GCS), Inflammation, Ventilation, Organ function, *APACHE* *II* Acute Physiology and Chronic Health Evaluation II, *CAR* C-reactive protein/Albumin ratio, *SOFA* Sequential Organ Failure Assessment, *NEWS* National Early Warning Score, *SAPS* *II* Simplified Acute Physiology Score, *RDW* Red Cell Distribution Width

The A-VIVO score, along with APACHE II, SOFA, SAPS II, and NEWS scores, was significantly higher in deceased patients compared to survivors (*p* < 0.001 for all).

Diabetes, hypertension, cerebrovascular disease, chronic respiratory disease, cardiovascular disease, and chronic kidney disease were significantly more frequent in patients who died (*p* < 0.05 for all). Mean arterial pressure (MAP) and Glasgow Coma Score (GCS) values, reflecting hemodynamic and neurological status, were significantly worse in patients who died (*p* < 0.001).

Laboratory parameters reflecting organ dysfunction and inflammation (creatinine, albumin, total bilirubin, C-reactive protein, CRP/albumin ratio, lactate, neutrophil-lymphocyte ratio, and hematological parameters) were found to be significantly more impaired in deceased patients compared to surviving patients (*p* < 0.001 for all).

**Fig. 2:**
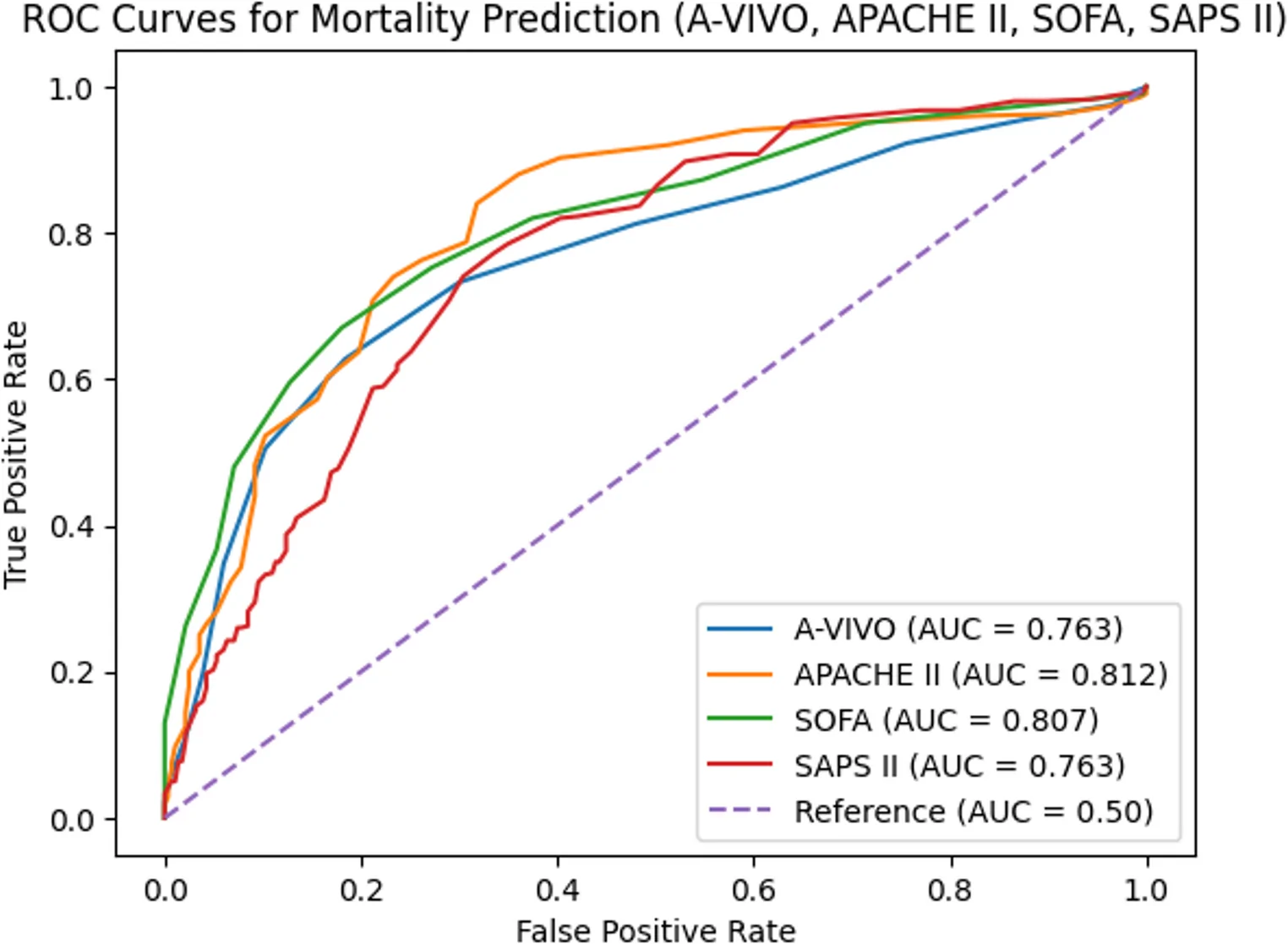
Receiver operating characteristic (ROC) curves of A-VIVO, APACHE II, SOFA, and SAPS II scores for mortality prediction

The predictive performance of A-VIVO score and traditional disease severity scores for 30-day mortality prediction was evaluated using ROC curve analysis (Table 3; Fig. 2). The discriminative power of the A-VIVO score was found to be AUC = 0.763 (95% CI: 0.727–0.799). APACHE II and SOFA scores had statistically higher AUC values compared to A-VIVO (*p* = 0.034 and *p* = 0.007, respectively), while there was no significant difference between A-VIVO and SAPS II (*p* = 0.997).

**Table 3:** Area under the ROC curve (AUC) for mortality prediction

Score	AUC	95% CI	*p* value vs. A-VIVO
A-VIVO	0.763	0.727–0.799	–
APACHE II	0.812	0.778–0.845	0.034
SOFA	0.807	0.775–0.839	0.007
SAPS II	0.763	0.726–0.800	0.997

A-VIVO Score; Age, Vital signs (MAP and GCS), Inflammation, Ventilation, Organ function, *APACHE* *II* Acute Physiology and Chronic Health Evaluation II, *SOFA* Sequential Organ Failure Assessment, *SAPS* *II* Simplified Acute Physiology Score

Using the Youden index, the optimal cutoff value for predicting mortality with an A-VIVO score was determined to be ≥ 9. At this threshold, the sensitivity of the A-VIVO score was calculated as 62.8%, the specificity as 81.6%, the positive predictive value as 82.8%, and the negative predictive value as 60.8%. The positive and negative odds ratios were 3.42 and 0.46, respectively. Logistic regression analysis showed that each 1-point increase in A-VIVO score was associated with approximately a 40% increase in mortality risk (OR = 1.40; 95% CI: 1.32–1.49; *p* < 0.001) (Table 4).

**Table 4:** Optimal cut-off value, diagnostic accuracy, and logistic regression analysis of the A-VIVO score for mortality prediction

Measure	Value
Optimal cut-off (Youden index)	≥ 9
Sensitivity (%)	62.8
Specificity (%)	81.6
Positive predictive value (PPV, %)	82.8
Negative predictive value (NPV, %)	60.8
Positive likelihood ratio (LR+)	3.42
Negative likelihood ratio (LR−)	0.46
Odds ratio per 1-point increase	1.40
95% confidence interval	1.32–1.49
*p* value	< 0.001

The optimal cut-off value was determined using the Youden index. Odds ratios were calculated using univariable logistic regression analysis with mortality as the dependent variable

In the APACHE II-like risk stratification based on the A-VIVO score, it was observed that mortality rates increased gradually with the score ranges (Table 5). The observed mortality rate was 31.0% in patients with an A-VIVO score of 0–4, while it reached 87% in patients with a score of 10–16. Agreement was found between the estimated mortality rates obtained from the logistic regression model and the observed mortality rates.

**Table 5:** Observed and predicted mortality according to A-VIVO score categories

A-VIVO risk group	A-VIVO score range	*n*	Deaths (*n*)	Observed mortality % (95% CI)	Predicted mortality (%)
Low risk	0–4	100	31	31.0 (22.8–40.6)	23.7
Intermediate risk	5–9	352	167	47.4 (42.3–52.7)	53.1
High risk	≥ 10 (10–16)	231	202	87.4 (82.8–91.1)	82.4

**Fig. 3:**
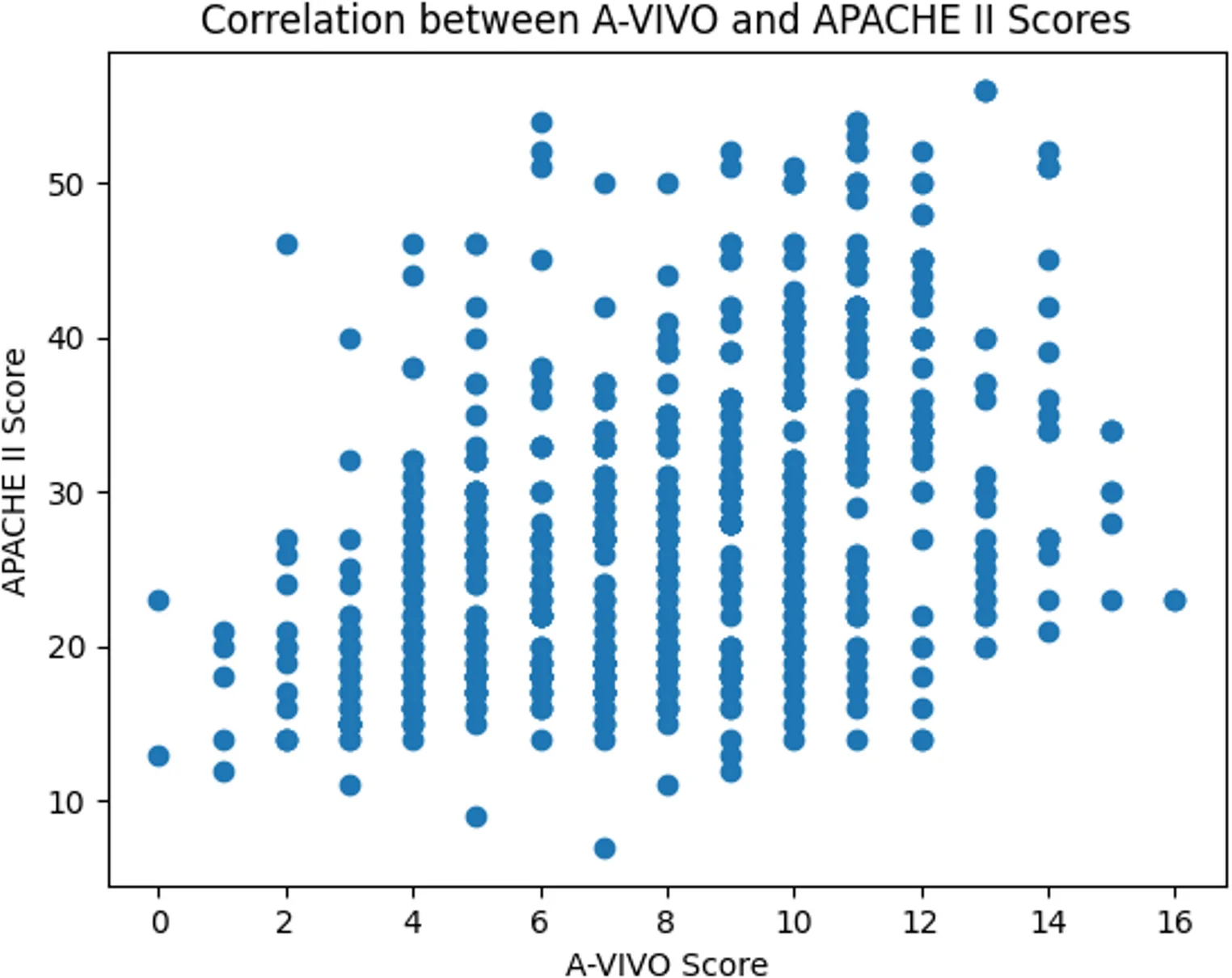
Scatter plot showing the correlation between A-VIVO and APACHE II scores

The relationship between the A-VIVO score and the APACHE II score was evaluated using Spearman correlation analysis. A moderately positive correlation was found between the two scores (Spearman *r* = 0.45; *p* < 0.001) (Table 6; Fig. 3).

**Table 6:** Spearman correlation analysis

Scores compared	Spearman’s *r*	*p* value
A-VIVO vs. APACHE II	0.45	< 0.001

A-VIVO Score; Age, Vital signs (MAP and GCS), Inflammation, Ventilation, Organ function, *APACHE* *II* Acute Physiology and Chronic Health Evaluation II

## Discussion

This study evaluated the prognostic performance of the A-VIVO score for 30-day mortality prediction in patients with sepsis admitted to the intensive care unit. The main findings demonstrate that the A-VIVO score is significantly associated with mortality and shows acceptable discriminative performance, with results comparable to SAPS II and close to APACHE II and SOFA, despite relying on fewer and more readily available parameters.

These findings suggest that the A-VIVO score may serve as a practical tool for 30-day mortality risk stratification in patients with sepsis, particularly given its reliance on routinely available clinical parameters.

In line with this, the observed mortality rate in the present study was 58.6%, which is higher than the rates commonly reported in the literature. This difference may be explained by the inclusion of critically ill patients in an intensive care setting, the presence of septic shock cases, the single-center design, and the high burden of comorbidities in the study population.

Scoring systems such as APACHE II, SOFA and SAPS II have been widely used for mortality prediction for many years [4, 9, 12]. However, these scores have common disadvantages. APACHE II and SAPS II require a large number of physiological and laboratory parameters, their calculations are time-consuming, and some variables are not routinely measured in all centers [4]. While the SOFA score reflects organ dysfunction well, it may not always have sufficient discriminatory power to predict 30-day mortality alone [9, 13].

In addition to traditional scoring systems, simplified tools such as qSOFA, NEWS, and MEWS have been developed for rapid bedside assessment. While these tools are easy to apply, their predictive performance for mortality has been reported to be variable and, in some cases, inferior to more comprehensive scoring systems [13, 14].

The A-VIVO score was developed to address these limitations by combining simplicity with clinically relevant laboratory and organ dysfunction parameters. Unlike qSOFA and similar tools, A-VIVO incorporates markers of inflammation, tissue hypoperfusion, and organ failure, while still maintaining ease of calculation.

Importantly, the A-VIVO score requires fewer parameters and simpler calculations compared to traditional scoring systems such as APACHE II and SAPS II, which enhances its usability in routine clinical practice.

The A-VIVO score was developed based on the fundamental components of sepsis pathophysiology. The parameters comprising the score are age, MAP, GCS, NLR as an indicator of inflammation, respiratory support requirement, and creatinine, total bilirubin, and lactate levels reflecting organ dysfunction. Each of these parameters is a variable significantly associated with sepsis mortality in the literature.

Age is an independent risk factor in sepsis prognosis, and it is known that the risk of mortality increases significantly with age [15, 16]. MAP, which reflects hemodynamic status, is critical for maintaining tissue perfusion in septic shock, and low MAP values have been shown to be associated with poor prognosis [6, 17]. GCS has long been used as a simple but powerful indicator of central nervous system involvement and global disease severity in sepsis [10, 18, 19].

NLR, representing inflammation in the A-VIVO score, is an inexpensive and objective parameter that can be easily obtained from a complete blood count. It has previously been shown that NLR is associated with mortality in critically ill sepsis patients and has independent prognostic value [7, 20, 21]. In this respect, NLR is a marker not included in classical scores but gaining increasing importance in clinical practice.

Creatinine and total bilirubin were included to reflect renal and hepatic dysfunction, respectively. lactate level is a strong indicator of tissue hypoperfusion and accumulated metabolic impairment. Lactate is a well-established independent predictor of mortality in sepsis [8]. The combined evaluation of these parameters allows for early detection of multi-organ dysfunction.

In this study, the A-VIVO score demonstrated an AUC of 0.763, indicating acceptable discriminative ability. This level of performance is generally considered sufficient for clinical risk stratification and is comparable to that of established scoring systems in similar patient populations.

However, APACHE II and SAPS II have a much larger number of parameters and complex calculation processes [4, 12, 13]. The fact that A-VIVO shows similar prognostic performance with fewer parameters suggests that the score may offer a practical advantage in terms of clinical efficiency and applicability.

The A-VIVO ≥ 9 cutoff value, determined by the Youden index, provided clinically significant risk discrimination. The high specificity, in particular, suggests that A-VIVO can be useful in identifying patients at high risk of mortality. Logistic regression analysis showed that each 1-point increase in the A-VIVO score approximately increased the risk of mortality by 40%, supporting the idea that the selected parameters of the score are clinically significant and complementary.

The moderate positive correlation found between A-VIVO and APACHE II indicates that both scores reflect a similar burden of disease severity. However, the fact that A-VIVO is based solely on routine intensive care data makes it a practical alternative, especially in clinical settings where time is limited and data access is restricted. This suggests that A-VIVO can be positioned as a complementary tool for early and rapid risk assessment, rather than a replacement for classical scores.

This study has several limitations. First, its retrospective and single-center design may limit the generalizability of the findings. Second, potential selection bias and missing data may have influenced the results. In addition, equal weighting was assigned to all variables included in the A-VIVO score to enhance simplicity and bedside applicability; however, the lack of data-driven weighting based on regression coefficients may have limited the predictive performance of the model. Furthermore, the A-VIVO score was developed and evaluated within the same cohort, and no internal validation methods such as bootstrapping or cross-validation were performed, which may increase the risk of overfitting. Finally, external validation in independent and prospective cohorts is required to confirm the generalizability of the score.

## Conclusion

In conclusion, the A-VIVO score, which has been shown to be significantly associated with mortality in sepsis patients, consists of a rational combination of parameters reflecting hemodynamic status, level of consciousness, inflammation, and organ dysfunction. Its comparable prognostic performance to SAPS II, and close to APACHE II and SOFA, despite requiring fewer parameters, makes A-VIVO a simple, rapid, and applicable risk assessment tool in clinical practice.

## Supplementary Information

Below is the link to the electronic supplementary material.

Supplementary Material 1
